# Downregulation of Foxo3 and TRIM31 by miR-551b in side population promotes cell proliferation, invasion, and drug resistance of ovarian cancer

**DOI:** 10.1007/s12032-016-0842-9

**Published:** 2016-10-14

**Authors:** Zhentong Wei, Yan Liu, Yishu Wang, Yandong Zhang, Qinghua Luo, Xiaxia Man, Feng Wei, Xiaowei Yu

**Affiliations:** 1 Department of Oncologic Gynecology, Prenatal Diagnosis Center, The First Hospital of Jilin University, Changchun, 130021 Jilin China; 2 Department of Hepatobiliary and Pancreas Surgery, The First Hospital of Jilin University, Changchun, 130021 Jilin China; 3 The Key Laboratory of Pathobiology, The Ministry of Education, Norman Bethune College of Medicine, Jilin University, Changchun, 130021 Jilin China; 4 Institute of Zoonotic Disease, Jilin University, Changchun, 130021 Jilin China; 5 Genetic Engineering Laboratory of People’s Liberation Army, The Eleventh Institute of Academy of Military Medical Sciences of People’s Liberation Army, Changchun, 130021 Jilin China; 6 Department of Rheumatology, The First Hospital of Jilin University, Changchun, 130021 Jilin China

**Keywords:** Ovarian cancer, Side population of cancer cells, miR-551b, Cell proliferation, Cell invasion, Drug resistance

## Abstract

Ovarian cancer (OVCa) stem cells are associated with tumor growth, metastasis, and recurrence, which are driving forces behind a majority of the OVCa-related mortality. This subpopulation of cancer cells are characterized by uncontrolled proliferation, high invasiveness, and resistance against the current platinum-based therapy. Thus, targeting OVCa cancer stem cells has been focused in recent therapeutic development. Isolation and purification of cancer stem cells are, however, challenging for the lack of sensitive and specific markers. In this study, we demonstrated that miR-551b was upregulated in OVCa stem cells, by using a quantitative PCR array, correlating with the pathological grades of this malignancy. In vitro experiments indicated that miR-551b promoted the proliferation, invasion, and chemoresistance of OVCa cells and cancer stem cells. Further analysis suggested that miR-551b functioned through the suppression of Foxo3 and TRIM31, two important tumor suppressors. In support of this, our in vivo experiments using mouse xenograft models showed that inhibiting miR-551b significantly increased the susceptibility of OVCa cells to cisplatin and prolonged the survival of the host mice. In conclusion, our study suggested miR-551b as a potential biomarker for OVCa stem cells and explored its functional mechanism, providing a potential therapeutic target for future drug development.

**Electronic supplementary material:**

The online version of this article (doi:10.1007/s12032-016-0842-9) contains supplementary material, which is available to authorized users.

## Introduction

Ovarian cancer (OVCa) is the most common and one of the most lethal gynecological malignancies in the world [1]. The overall 5-year survival rate has been merely 28–40 % in the past few decades [2–4]. Primary OVCa, particularly high-grade serous carcinoma that accounts for majority of this malignancy, usually responds to the first-line platinum-based chemotherapy, but the disease frequently recurs in a more aggressive form and with increased chemoresistance [1]. Although extensive studies have been performed on the recurrence and chemoresistance of OVCa in the past few decades [5, 6], the clinical outcome has not improved substantially.

Cancer stem cells (CSCs) comprise of a small number of cells with stem cell features among the highly heterogeneous mixture of various populations of cells in tumor. Characteristically, they are poorly differentiated and self-renewable as normal stem cells and are thus capable of proliferating continuously and re-initiating tumor in vivo. CSCs play a crucial role in maintaining tumor heterogeneity and promoting cancer cell growth and metastasis [7, 8]. Moreover, they are generally less susceptible to chemotherapeutic drugs and likely the re-initiator of the recurrent cancer [9, 10]. Therefore, identifying this subpopulation of cells and eradicating them from patients may provide an ideal opportunity to cure this disease. Indeed, targeting CSCs by interrupting PML-RARα degradation and BMP4 function in animal models has significantly enhanced the regression of acute promyelocytic leukemia and brain cancer [11, 12]. Ovarian cancer is believed to be a stem cell disease. It may originate from ovarian surface epithelium stem cells or fallopian tube epithelium [13–16]. A number of biomarkers have been recommended although definitive and commonly accepted markers remain elusive [17, 18]. Recently, a few studies isolated a side population (SP) of cells based on their exclusion of Hoechst 33342 fluorescence. These cells demonstrate extensive features of CSCs with respect to their proliferation, tumorigenicity, migration, and chemoresistance, suggesting that they can be ideal subjects in CSC studies [19]. Particularly in OVCa, independent studies have shown that SP cells isolated from fresh tumors and established cell lines are enriched with tumor-initiating cells with CSC characteristics and are highly chemoresistant [20, 21]. In our previous study, we isolated SP cells from a primary OVCa cell line established from OVCa patient ascites and characterized their self-renewal, differentiation, and tumorigenicity [22].

microRNAs refer to a group of small noncoding RNAs which participate into the posttranscriptional gene expression control by inhibiting translation or accelerating mRNA degradation [23]. Aberrant expression of microRNAs frequently observed in multiple malignancies is associated with tumor progression [24, 25]. A good example of these is miR-551b, which is upregulated in lung squamous cell carcinoma (SCCs) and prostate cancer, and mediates tumor development and progression [26, 27]. Elevated miR-551b in OVCa enhances the resistance of cancer cells to anoikis by upregulating STAT3 and c-KIT [28].

Accumulating evidence suggests that microRNAs are pivotal for the self-renewal of CSCs and cancer metastasis and chemoresistance [29–31]; however, the underlying mechanisms are yet to be fully understood. In this study, we aimed to screen for microRNAs, which were abnormally expressed in the SP cells of OVCa in comparison with the non-SP cells. We identified miR-551b as one of the most significantly elevated microRNAs in the SP cells in comparison with the non-SP cells in OVCa. Using an miR-551b mimic or an specific inhibitor, we explored the roles of miR-551b in cell proliferation, invasion, and susceptibility to cisplatin and identified the downstream effectors. In a mouse xenograft model, miR-551b was targeted to confirm the regulatory roles of miR-551b in OVCa progression and characterize the potentiality of targeting miR-551b in future therapeutic development.

## Materials and methods

### Patient samples

Benign and malignant OVCa tissues and ascites fluids were collected from Department of Obstetrics and Gynecology, the First Hospital of Jilin University with written consents from all patients. All protocols involving human specimens were approved by the Human Ethics Committee of the First Hospital of Jilin University. Detailed diagnostic and pathological reports were collected for all patients, and none of them had been previously treated with chemotherapy.

### Cell culture, isolation of SP cells, and transfection

Primary OVCa cells were isolated from the ascites of an ovarian serous cystadenocarcinoma patient and maintained as previously described [22]. Briefly, primary ascitic cells were harvested by centrifugation at 300*g* for 5 min, and red blood cells removed by 1× BD lysis buffer (BD Biosciences, Franklin Lakes, NJ) on ice for 1 min, followed by centrifugation at 300*g* for 3 min. Primary cells were cultured for 3 weeks in a Dulbecco’s modified Eagle’s medium (DMEM, Invitrogen, Carlsbad, CA) supplemented with 10 % fetal bovine serum (FBS), and the floating cells were collected and re-cultured. This ascites-derived OVCa cell line was established by continuous propagation. HEK293T cells were grown in DMEM supplemented with 10 % FBS (Invitrogen). All cells were cultured at 37 °C in a humidified atmosphere with 5 % CO_2_ in the presence of penicillin (100 units/ml) and streptomycin (100 units/ml). The cisplatin–resistant cell line was established as described previous [32]. Briefly, cisplatin-sensitive SK-OV-3 and 8910 cells parental cells were exposed to gradually increasing concentration of cisplatin (LC laboratories) from the initial 1 μM to final 60 μM over a 6-month period.

To isolate the SP cells, the primary ascites-derived OVCa cells were trypsinized, pelleted, and re-suspended at 1.0 × 10^6^ cells/ml in DMEM containing 2 % flow cytometry staining buffer (BD Biosciences) and incubated at 37 °C for 10 min. The cells were then labeled with 5 μg/ml Hoechst 33342 dye (Invitrogen) at 37 °C for 80 min, followed by counterstaining with 1 μg/ml propidium iodide. A total of 100,000 cells were sorted on a BD Influx system, and data were processed by BD FACSDiva software (version 6.1.1, BD Biosciences).

Cells were transfected in an Opti-MEM medium (Invitrogen) with miR-551b mimic, miR-551b inhibitor, scramble RNA (GeneCopoeia, Rockville, MD) or psiCHECK-2 plasmid (Promega, Madison, WI) using Lipofectamine 2000 (Invitrogen), following the manufacturer’s instructions. Cells were collected and analyzed 48 h after transfection.

### Cell proliferation assay

Cells were seeded into 96-well plates at 3000 cells/well and cultured for 24 h. The medium was then replaced with 10 µl of cell counting kit (CCK)-8 reagent (Dojindo Laboratories, Kumamoto, Japan) and 100 µl of HEPES-buffered DMEM medium (Invitrogen) containing 10 % FBS. After another 2.5 h of culture at 37 °C, cell viability was assessed by measuring the absorbance of individual wells at 450 nm. Five replicates were performed for each group.

### Colony formation assay

Capacities of cells to form colonies were determined by two approaches. In the monolayer colony formation assay, 500 single cells were seeded into 35-mm dishes and cultured for 10 days with medium refreshed every 3 days. At measurement, the medium was discarded, cells were stained with crystal violet (0.1 % in 20 % methanol) and imaged under a SZX12 phase-contrast microscope (Olympus, Tokyo, Japan), and colonies counted.

Soft agar colony formation assay was performed following a protocol used elsewhere with limited modifications. Briefly, 500 µl of 0.5 % agar (Sigma-Aldrich, St. Louis, MO) prepared in appropriate cell culture medium was aliquoted into 24-well plates (500 µl/well) and allowed to solidify. On the top of this, 500 µl of cell suspension at 2.66 × 10^2^ cells/ml prepared in 0.3 % agar was added. The cells were cultured for 3 weeks, with medium refreshed twice a week, before the colonies larger than 75 µm in diameter or containing more than 50 cells were counted under the microscope.

### RNA isolation and qPCR

RNA from cells and tissues was isolated with a Trizol reagent (Invitrogen) following the manufacturer’s instructions and used as templates in the synthesis of the first-strand complementary DNA using a TaqMan microRNA reverse transcription kit (Applied Biosystems, Foster City, CA). qPCR was performed in triplicate using a TaqMan universal PCR master mix (Applied Biosystems). The thermal cycling conditions included a 10-min denaturation at 95 °C followed by 35 cycles of 15-s denaturation at 95 °C, 1-min annealing at 60 °C, and 45-s extension at 72 °C.

### Western blotting

Total proteins from cells and tissues were isolated with a RIPA buffer (Cell Signaling Technology, Danvers, MA) in the presence of a protease inhibitor cocktail (Thermo Scientific), separated by sodium dodecyl sulfate-polyacrylamide gel electrophoresis (SDS-PAGE), and transferred onto polyvinylidene fluoride (PVDF) membranes. After blocking with 5 % bovine serum albumin for 1 h at room temperature, the membranes were incubated overnight at 4 °C with primary antibodies against Foxo3, TRIM31, and GAPDH (Cell Signaling Technology) and then for 1 h at room temperature with appropriate horseradish peroxidase-conjugated secondary antibodies (Abcam, Cambridge, UK). The signals were detected with an enhanced chemiluminescence detection kit (Thermo Scientific).

### Construction of the plasmids and dual luciferase reporter assay

The reverse complementary miR-551b (rcmiR-551b), the wild-type and mutant 3′-untranslated regions (UTR) of Foxo3 and TRIM31 were synthesized by TaKaRa (Shanghai, China) and inserted into psiCHECK-2 or pcDNA 3.1 vectors. The restriction enzymes and T4 DNA ligase were purchased from New England Biolabs (Ipswich, MA). The sequences were confirmed by Sanger sequencing at Comate Bioscience (Changchun, China).

The dual luciferase reporter assay was performed using HEK293T cells in 24-well plates. The cells were co-transfected with 0.8 µg/well of plasmid and 30 nM of miR-551b mimic or its inhibitor and incubated for 24 h. The samples were then analyzed using a dual luciferase reporter assay kit (Promega), and the assays were performed in triplicate and repeated three times.

### Cell cycling profiling

Cells were synchronized by serum starving for 24 h and then transfected with miR-551b mimic, inhibitor, or the scramble RNA. After 48 h, cells were detached, fixed in 70 % ethanol, and stained with propidium iodide before being analyzed for cell cycle profiles by flow cytometry using a FACSCanto II system (BD Biosciences). The results were processed using a FlowJo data processing software (FlowJo, Ashland, OR).

### Cell invasion assay

Cell invasion was assessed by using a BioCoat Matrigel invasion chamber (BD Biosciences). The 24-well plate was fit with inserts, which were sealed with 8-µm membranes at the bottom, and the inserts were pre-coated with Matrigel. To examine cell invasion, cells were serum starved for 24 h and added into the top chamber at 1 × 10^4^ cells in 100 µl serum free medium, while 600 µl medium with 1 % FBS was added to the bottom. After culturing for 12 h at 37 °C, the medium in the insert was decanted, and cells and Matrigel were carefully removed with a cotton swab. Cells attached to the outside of the insert were stained with DAPI and imaged and counted under a microscope. Ten regions of interest (magnification, 40×) were counted for each group.

### Mouse xenograft assay

All protocols using animals were approved by the Institutional Animal Care and Use Committee of Jilin University. Six-week-old female SCID mice (Shanghai Laboratory Animal Centre, Shanghai, China) were inoculated subcutaneously with 3 × 10^4^ SP cells premixed with Matrigel at a 1:1 ratio. After the tumors were established for 2 weeks, the mice were treated by intratumoral injection of 5 µl of 5 nM agomir, 20 nM antagomir, or saline twice a week for 4 weeks. In a second approach to examine the tumor growth in peritoneal cavity, the SP cells re-suspended in saline were injected intraperitoneally at 5 × 10^4^ cells/mouse, and the intraperitoneal administration of agomir or antagomir (twice a week for 4 weeks) began after the tumors were established.

Twenty mice were used for each group. Ten of them were used to generate the survival curve, whereas the other 10 were killed 6 weeks after inoculation. Tumor tissues were excised, weighed, and further used in qPCR and Western blotting analyses.

### Statistical analysis

Data are presented as mean ± standard error of the mean (SEM), which was obtained from a minimum of three repeats of each experiment. Data processing was performed using a GraphPad Prism 5 software (GraphPad, La Jolla, CA), and Student’s *t* test and one-way analysis of variance (ANOVA) were used to estimate the significance of difference, and *p* < 0.05 was considered significant.

## Results

### miR-551b is elevated in OVCa

**Fig. 1:**
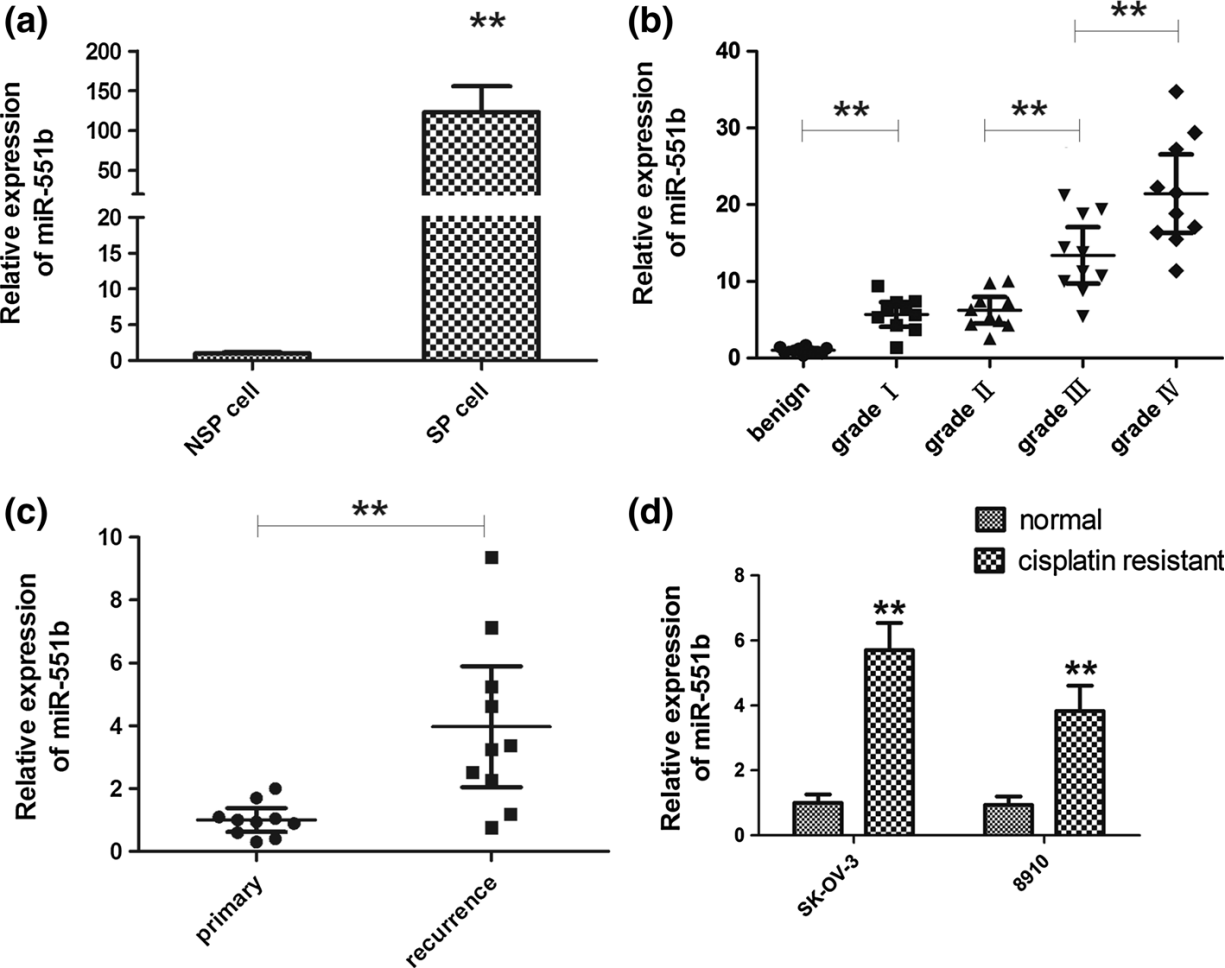
miR-551b is upregulated in the side population of an ascites-derived OVCa cell line, and its expression correlates with the stage, recurrence, and chemoresistance of OVCa. **a** qPCR analysis indicates a higher level of miR-551b in the side population of the ascites-derived OVCa cell line than the non-side population, **b** expression of miR-551b in ovarian serous cystadenocarcinoma tissues with varied levels of differentiation was examined by qPCR, in comparison with ovarian serous cystadenoma. Ten patients were included in each group, **c** expression of miR-551b in the primary (Stage III) and recurrent OVCa. Samples from 10 patients were examined in each group. **p < 0.01, **d** expression of miR-551b was compared between the cisplatin-sensitive and cisplatin-resistant OVCa cells. **p < 0.01

To identify microRNAs aberrantly expressed in the OVCa SP cells in comparison with the non-SP cells, the expression of 16 microRNAs was quantified by qPCR array analysis performed by Shanghai Biotechnology Corporation (Shanghai, China). In the results, miR-551b was one of the top 10 microRNAs that were upregulated in the OVCa SP cells compared to the non-SP population. This result was validated by a separate qPCR analysis for miR-551b (Fig. 1a). Consistent with this, significantly higher levels of miR-551b were detected in tissues from the advanced serous OVCa than the early stage (Fig. 1b), suggesting the association of miR-551b expression with OVCa progression. Furthermore, miR-551b was significantly upregulated in the recurrent serous OVCa compared to the primary tumors (Fig. 1c), and the cisplatin-resistant cells expressed more miR-551b than the susceptible cells (Fig. 1d), correlating with its overexpression in the stem cell-enriched SP cells, which are believed to drive tumor recurrence.

### miR-551b promotes the proliferation and invasion of OVCa cells

**Fig. 2:**
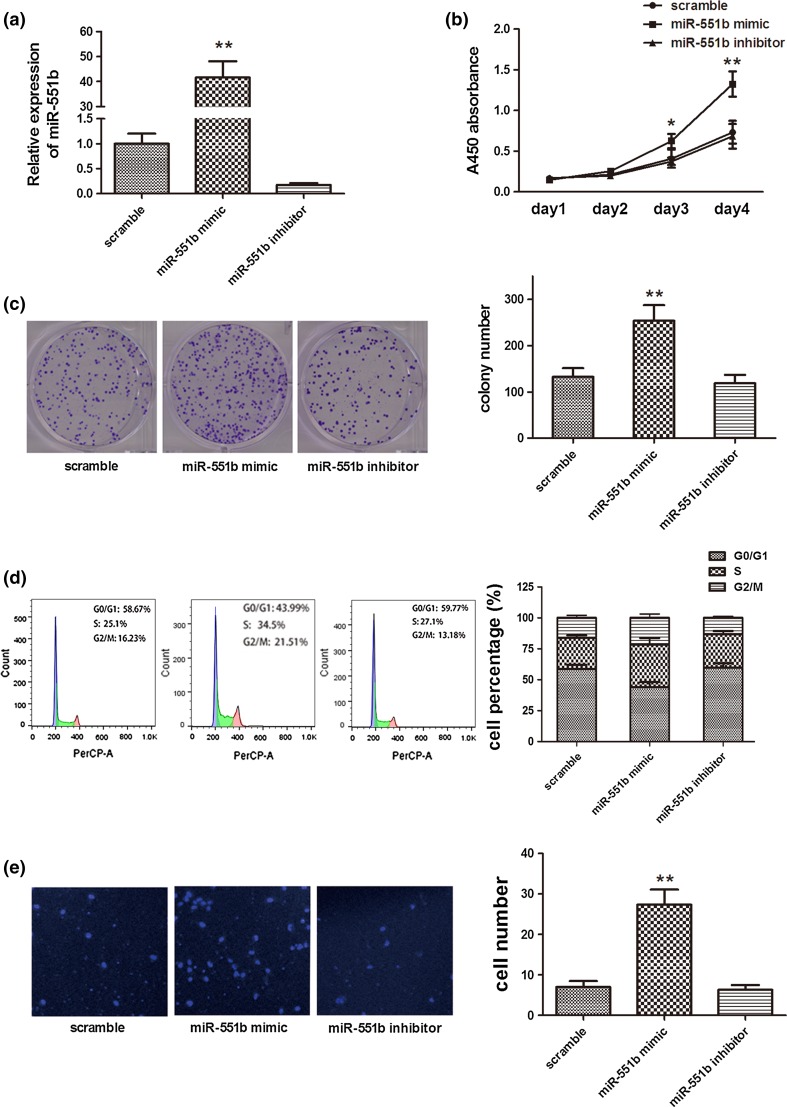
miR-551b promotes the proliferation and invasion of OVCa cell lines. **a** Expression of miR-551b was manipulated by transfecting cells with miR-551b mimic or miR-551b inhibitor, using scramble RNA as a control, and the expression of miR-551b was examined by qPCR, **b** miR-551b significantly enhanced cell proliferation as assessed by a CCK-8 assay, **c** monolayer colony formation of cells transfected with miR-551b mimic or miR-551 inhibitor was compared to the scramble control, **d** cell cycle profiles were determined by flow cytometry, and the results presented as percentages of cells in G0/G1, S, and G2/M phases, **e** cells with upregulated miR-551b expression showed enhanced invasion through Matrigel in a transwell system. Cells migrated through the Matrigel layer were stained with DAPI and imaged under a fluorescence microscope, *scale bar* 100 µm. Results are averaged from three repeats of all experiments. **p < 0.01; *p < 0.05

The cellular roles of miR-551b were characterized by in vitro assays using the primary OVCa cell line established in house. Cells were transfected with miR-551b mimic, miR-551b inhibitor, or the scramble RNA, and the changes in the expression of miR-551b were confirmed by qPCR (Fig. 2a). As a result, upregulating miR-551b expression led to significant increase in cell proliferation, while cells with miR-551b inhibitor showed no difference from the scramble transfected cells (Fig. 2b). Similar results were obtained in the monolayer colony formation assay (Fig. 2c). The enhancement of cell proliferation by miR-551b was supported by the profiling of the cell cycle. In Fig. 2d, flow cytometry analysis demonstrated that increased miR-551b reduced the cell population in the G1 phase while increasing that in the S phase, suggesting that this microRNA may promote the transition from G1 to S phase.

The invasion potential of cells transfected with miR-551b mimic, miR-551b inhibitor, or the scramble RNA was examined by a transwell system in which the inserts were pre-coated with a layer of Matrigel. The results indicated a significant increase in the number of cells migrating through Matrigel when the primary OVCa cells were transfected with miR-551b mimic, whereas those with miR-551b inhibitor showed no apparent difference, compared to the cells with the scramble control (Fig. 2e).

### miR-551b promotes the colony formation of the SP cells and confers them chemoresistance

**Fig. 3:**
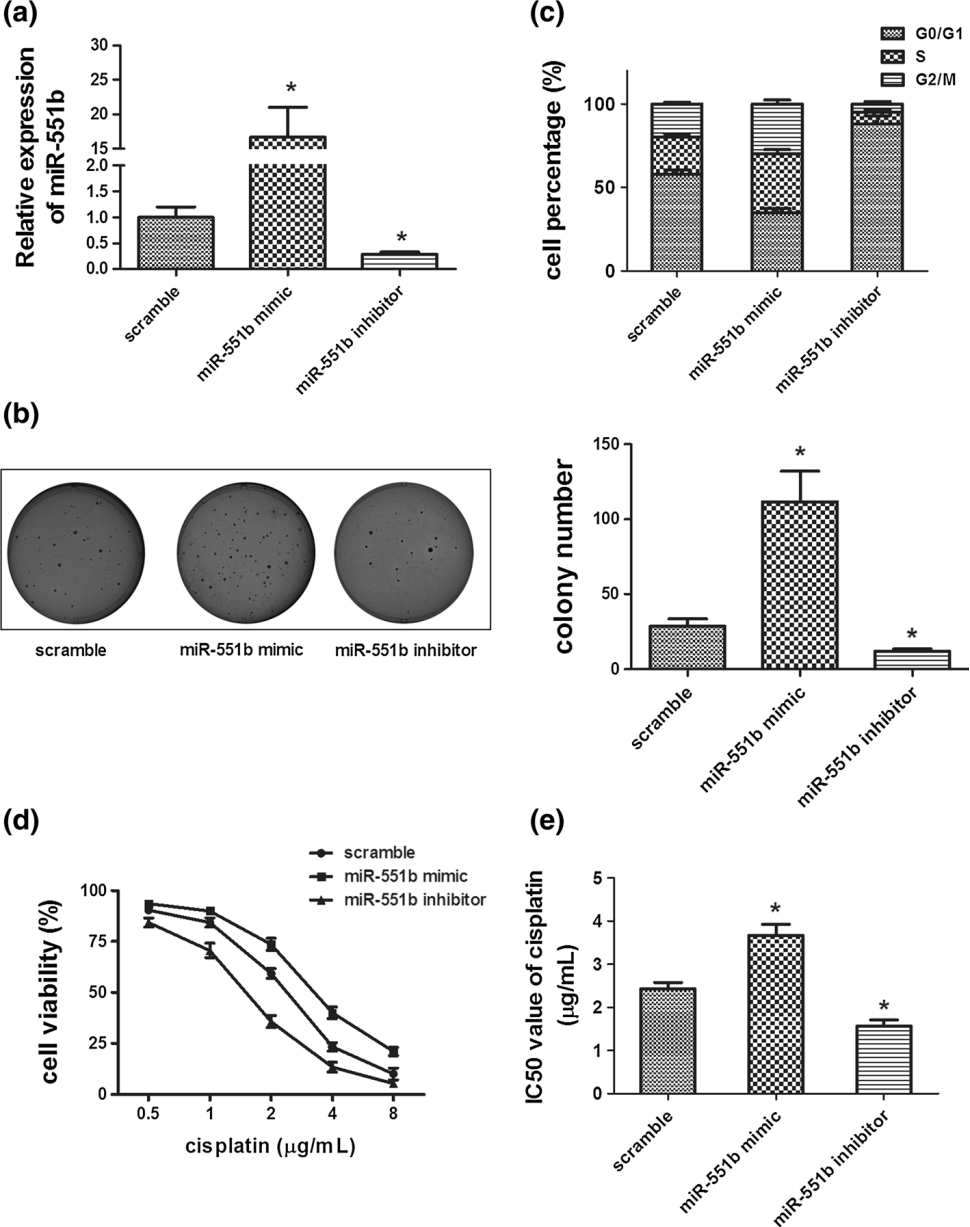
miR-551b increases the invasion of the SP cells from the ascites-derived OVCa cell line and enhances their chemoresistance. miR-551b expression was quantified by qPCR after the SP cells were transfected with miR-551b mimic, miR-551b inhibitor, or scramble RNA control (**a**). Colony formation of these cells in soft agar was then assessed (**b**), and cell cycle profiles determined by flow cytometry (**c**). The susceptibility of cells to chemotherapeutic drug was assessed by exposing them to 0.5–8 µg/ml cisplatin for 3 days and measuring their viability by a CCK-8 assay. IC_50_ values were calculated using GraphPad Prism 5.0 software. Data were averaged from three repeats of all experiments. *p < 0.05

To further dissect the functions of miR-551b in OVCa cells, we sorted out the SP cells from the ascitic OVCa cell population and performed the soft agar colony formation assay after manipulating their miR-551b expression (Fig. 3a). The results indicated that the SP cells transfected with miR-551b mimic produced more colonies than the scramble control (Fig. 3b). Consistently, SP cells transfected with miR-551b mimic had a smaller G1 but a larger S population of cells than those treated with the scramble RNA (Fig. 3c). More importantly, in these primary CSCs, inhibiting miR-551b expression using a sequence-specific inhibitor (Fig. 3a) dramatically reduced the colony formation capacity (Fig. 3b), and these data were correlated with a significantly larger G1 but a smaller S population of SP cells treated by miR-551b inhibitor (Fig. 3c).

Cancer stem cells are believed to be crucial in the chemoresistance of cancer due to their less responsiveness to the current platinum-based therapy [20, 33]. We thus further characterized whether miR-551b was involved in the chemoresistance of OVCa. The SP cells transfected with miR-551b mimic, miR-551b inhibitor, or the scramble RNA were exposed to a range of doses of cisplatin, and their viability assessed after 3 days. The results demonstrated a decreased susceptibility of cells treated with miR-551b mimic to cisplatin and an increased responsiveness of cells with miR-551b inhibitor (Fig. 3d). The IC_50_ values for cells with the scramble RNA, miR-551b mimic, and miR-551b inhibitor were 2.33, 3.41, and 1.49 µg/ml, respectively.

### Identification of the downstream targets of miR-551b

**Fig. 4:**
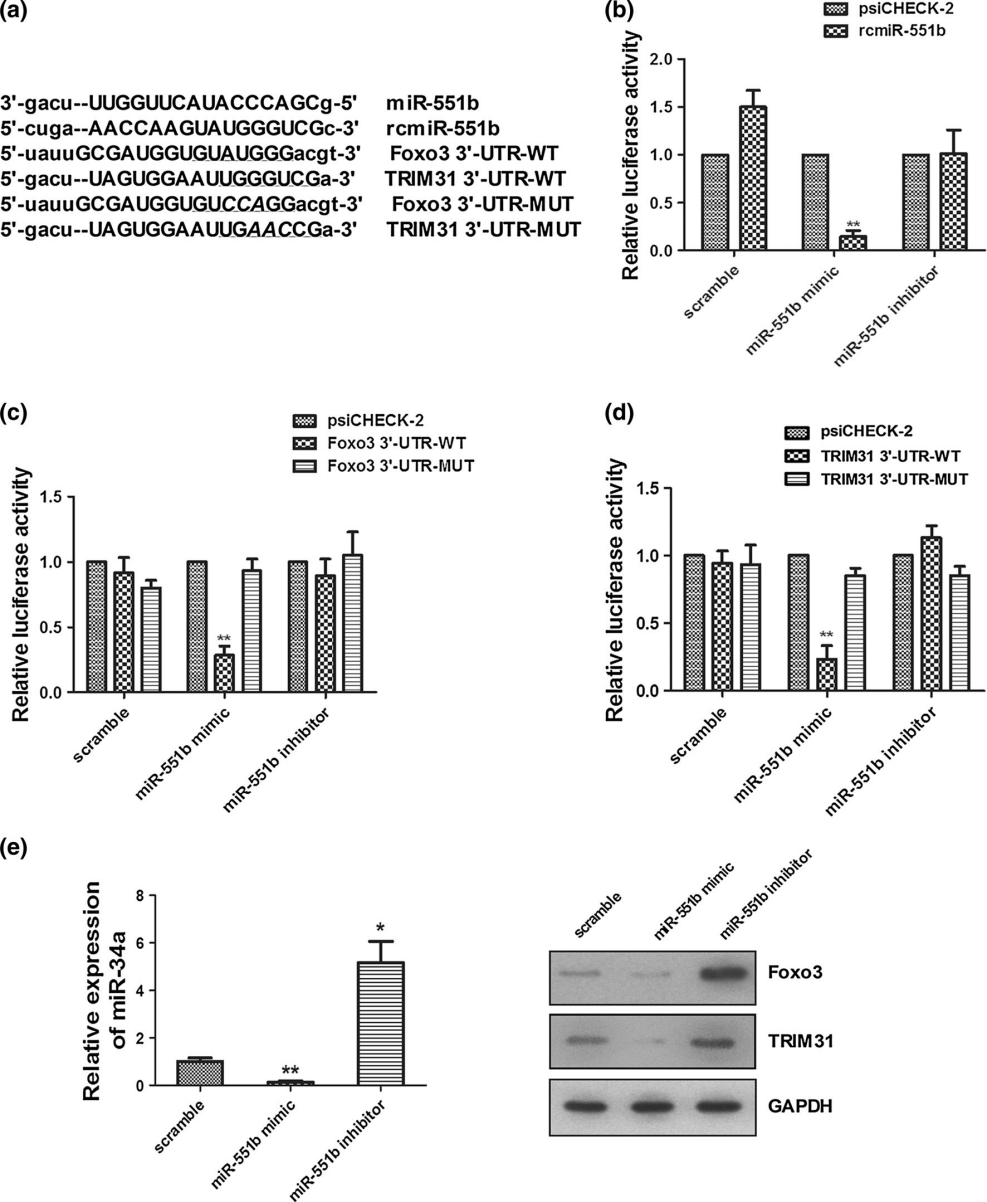
Foxo3 and TRIM31 are downstream targets of miR-551b. **a** Sequences of miR-551b, rcmiR-551b, and the potential targeting regions in the 3′ UTRs of Foxo3 and TRIM31 are shown. In the mutant forms of Foxo3 and TRIM31, mutated nucleotides are *underlined*, **b**–**d** association of miR-551b with rcmiR-551b, and the wild-type and mutant forms of 3′ UTRs of Foxo3 and TRIM31 in HEK293T cells were determined by a reporter assay. Data were averaged from three repeated experiments, **e** the mediation of Foxo3 and TRIM31 by miR-551b was confirmed by qPCR and Western blotting, respectively. **p < 0.01; *p < 0.05

We next asked how miR-551b executes its important functions in the proliferation, invasion, and chemoresistance of OVCa cells. Using three microRNA target prediction algorithms, including TargetScan, Pictar, and microRNA, we found that Foxo3 and TRIM31 were among the potential targets of miR-551b. The potential targeting sequences are shown in Fig. 4a. Foxo3 encodes one of the fork head family of transcription factors, which control the expression of multiple apoptosis regulating factors, such as p53 and p21 [34]. TRIM31 is a member of the tripartite motif family of proteins featured by RING finger, B-box, and coiled-coil domains. It is downregulated in multiple malignancies, correlating with tumor stage and metastasis [35], while its overexpression suppresses c-Src-induced cell growth by interacting with p52 [36].

To validate the mediation of Foxo3 and TRIM31 by miR-551b, we performed a dual luciferase reporter assay to assess the interaction of miR-551b with the wild-type and mutant 3′-UTRs of Foxo3 and TRIM31 using the empty psiCHECK-2 vector and rcmiR-551b as controls (Fig. 4b). The results showed that miR-551b mimic significantly inhibited the expression of Foxo3 and TRIM31, while miR-551b inhibitor led to no obvious change (Fig. 4c, d). These results were supported by qPCR and Western blotting analyses of TRIM31 and Foxo3, respectively, using the SP cells from OVCa (Fig. 4e). When the targeting sequences in the 3′-UTRs were mutated, the miR-551b-mediated effects were completely abolished (Fig. 4c, d), suggesting the interaction of miR-551b with the 3′-UTRs of Foxo3 and TRIM31.

To define whether Foxo3 and TRIM31 are involved in the miR-551b-mediated cellular functions, Foxo3 or TRIM31 was overexpressed in OVCa cells in the presence or absence of miR-551b mimic (Supplemental Fig. 1A). Cell proliferation assay demonstrated that miR-551b significantly promoted, consistently with our data in Fig. 2b, while exogenous Foxo3 and TRIM31 inhibited cell proliferation (Supplemental Fig. 1B, C). Importantly, both Foxo3 and TRIM31 apparently abrogated the induction of proliferation by miR-551b when either was co-transfected with miR-551b (Supplemental Fig. 1B, C). Flow cytometry analysis showed that the cells were arrested at G0/G1 phase when Foxo3 or TRIM31 was overexpressed (Supplemental Fig. 1D). The two proteins counteracted against miR-551b and negated the increase in S phase cells population caused by this microRNA (Supplemental Fig. 1D). Moreover, exogenously expressed Foxo3 or TRIM31 also overturned the increase in cell invasion induced by miR-551b (Supplemental Fig. 1E). These data suggested that miR-551b regulates the proliferation and invasion of the OVCa SP cells through its inhibitive effect on the expression of Foxo3 and TRIM31.

### Targeting miR-551b increases the susceptibility of mouse xenografts to cisplatin

**Fig. 5:**
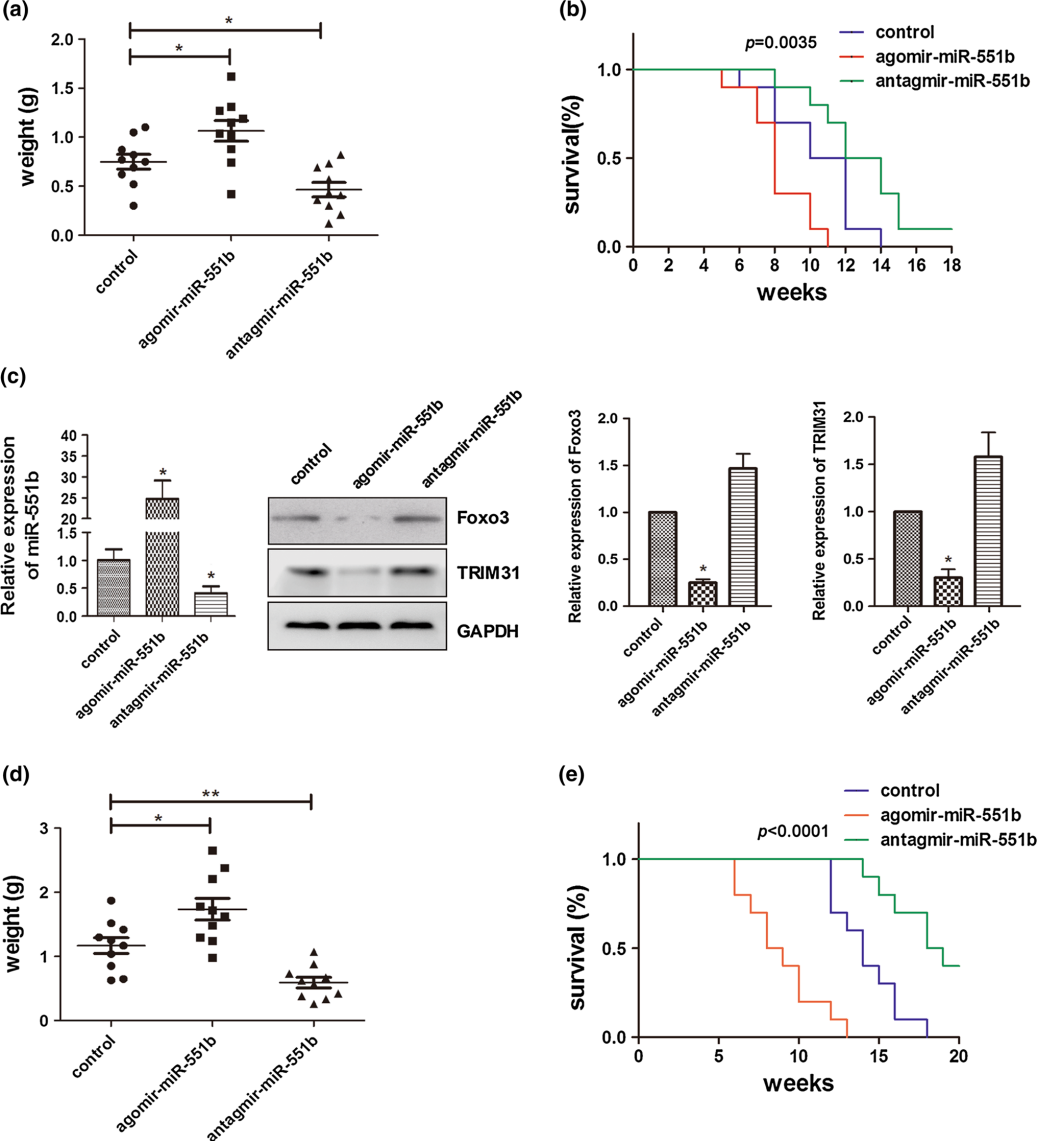
Blocking the expression of miR-551b inhibits the growth of ovarian tumors in vivo. In the subcutaneous mouse xenograft models, the tumor burden in mice was significantly increased by the 4-week treatment of miR-551 agomir, but reduced by miR-551b antagomir (**a**), and correlated with these, mice injected with miR-551b agomir had poorer survival (*p* = 0.0035), while those with miR-551b antagomir showed better survival in comparison with the control mice (**b**). Expression of miR-551b, Foxo3 and TRIM31 in the xenografts was assessed by qPCR and Western blotting (**c**). In the intraperitoneal mouse xenograft model, mice were injected intraperitoneally with SP cells and were treated for 4 weeks with miR-551b agomir, miR-551b antagomir, or the vehicle, in the presence of 25 mg/kg cisplatin (intraperitoneal delivery). Tumor burdens in mice at harvest were compared (**d**), and survival curves graphed (**e**). **p < 0.01; *p < 0.05

Mouse xenograft model was employed to confirm our in vitro observations, which suggested key roles of miR-551b in the proliferation and invasion of OVCa cells. The SP cells were inoculated subcutaneously into the flanks of SCID mice, and the established tumors were treated by intratumoral injection of agomir or antagomir of miR-551b to up- or downregulate miR-551b levels. At harvest after 4 weeks of treatment, miR-551b agomir increased the tumor burden by 42 %, compared to the controls which were injected with saline, while miR-551b antagomir decreased the tumor burden by 38 % (Fig. 5a). Corresponding to this, mice treated with miR-551b agomir survived for a shorter period than the control, while the treatment with miR-551b antagomir prolonged the survival (Fig. 5b).

In support of the mediation of Foxo3 and TRIM31 by miR-551b observed in vitro, Western blotting analysis revealed that the expression of Foxo3 and TRIM31 was inhibited in tumors treated by miR-551b agomir and enhanced in those by miR-551b antagomir (Fig. 5c). The data suggested that the mediation of Foxo3 and TRIM31 by miR-551b was occurring in vivo and might play a key role in the observed impacts of miR-551b on tumor growth (Fig. 5a).

Our in vitro data in Fig. 3d suggested that miR-551b mediated the resistance of the SP cells against cisplatin. We therefore tested whether this conclusion stands in an in vivo model. The freshly isolated SP cells were injected intraperitoneally into SCID mice and allowed to form tumors. Mice were treated by intraperitoneal administration of miR-551b agomir, miR-551b antagomir, or saline, together with 25 mg/kg cisplatin. When the mice were killed after 4 weeks of treatment, higher tumor burdens were measured in mice co-injected with miR-551b agomir and cisplatin than those with saline and cisplatin (Fig. 5d). On the other hand, mice co-administrated with miR-551b antagomir and cisplatin grew significantly fewer tumors (Fig. 5d), and as a result, these mice survived much longer periods than the other two treatment groups (Fig. 5e).

## Discussion

In this study, we screened for microRNAs aberrantly expressed in the SP cells isolated from an ascites-derived OVCa cell line in comparison with the non-SP cancer cells. miR-551b was identified as one of the top 10 upregulated microRNAs in the SP cells. Further in vitro and in vivo assays suggested that miR-551b mediated the proliferation, invasion, and chemoresistance, likely through its control over the expression of Foxo3 and TRIM31.

Majority of the experiments in the study were performed using the SP cells originated from malignant OVCa ascites, which is associated with the transcoelomic metastases of OVCa cells [37, 38]. The SP populations of cells in many cancers demonstrate stem cell features although contradictive results have also been reported for certain types of cancer. The enrichment of CSCs in the SP population of OVCa cells has been confirmed by independent studies including ours [22, 39, 40]. The results from these studies establish that the SP cells of OVCa possess significant stem cell characteristics, such as cell differentiation, colony formation, tumorigenesis, and chemoresistance [22]. Therefore, the SP cells of OVCa can be a useful model in the development of therapeutic approaches targeting CSCs or cells with stem cell features, which are disputably the major reasons behind the progression and the acquired chemoresistance of many malignancies [41].

Emerging evidence suggests a critical role of microRNAs in OVCa progression. Upregulation of the miR-200 family microRNAs predicts poor progression-free and overall survivals of OVCa patients [42]. On the other hand, microRNAs can be tumor suppressive. miR-7 reverses epithelial–mesenchymal transition by inactivating AKT/ERK1/2 via EGFR and inhibits OVCa metastasis [43]. miR-9 inhibits the proliferation, migration, and invasion of serous OVCa cells by blocking TLN1-mediated FAK/AKT pathway [44]. In addition, expression of miR-496, miR-152, miR-422b, and miR17-3p correlates with acquired cisplatin resistance [45]. The current study demonstrates that miR-551b is over 100-fold higher in the SP cells than in the non-SP population of cells, implicating that this microRNA is required to maintain the phenotypic features of the SP cells. Indeed, miR-551b upregulates STAT3 and c-KIT and enhances the resistance of ovarian tumor cells to anoikis [28]. This mechanism may extend to other anchorage-free settings since miR-551b expression is also enhanced in the circulating prostate cancer cells [27]. Further studies are needed to define whether miR-551b is involved in other processes important for OVCa metastases, such as spheroid formation and the colonization of OVCa cells on the surfaces of adjacent organs in the peritoneal cavity [27, 46].

In solid ovarian tumors, miR-551b expression correlates with tumor grades (Fig. 1b), suggesting its close association with cancer progression. This is consistent with the association of miR-551b expression with the survival of 296 OVCa patients, as demonstrated by previous microRNA profiling [47]. In addition, miR-551b expression is elevated in the recurrent OVCa compared to the primary disease (Fig. 1c) although its functional importance in OVCa recurrence is unknown. In lung cancer cells, miR-551b inhibits the expression of catalase and enhances the accumulation of reactive oxygen species and the expression of mucin-1, contributing to the acquired resistance to apoptosis and chemotherapy [48].

Our data support significant roles of miR-551b in the proliferation and invasion of the ascitic SP cells in vitro and the growth of tumor xenografts in mice (Fig. 5). This is likely through the suppression of Foxo3 and TRIM31 expression as shown by the binding of miR-551b to the UTRs of Foxo3 and TRIM31 transcripts (Fig. 4) and the reversing of the miR-551 mimic-induced phenotype by exogenous Foxo3 and TRIM31 (Supplemental Fig. 1). Foxo3 is a known regulator of p53 and p21 [34], exhibiting a tumor-suppressive role in high-grade pelvic serous carcinogenesis [49]. The observed shifts of the SP cells between G1 and S phases of the cell cycle in response to the manipulation of miR-551b expression are consistent with the known functions of Foxo3 in cell cycle progression [34]. TRIM31 is aberrantly expressed in multiple cancers. It is downregulated in lung cancer with tumor stage [35], whereas its elevation in gastric carcinoma leads to inhibition of cell proliferation [50]. At molecular level, TRIM31 interacts with p52 and regulates Src kinase [50]. Our results suggest a tumor-suppressive role of TRIM31 in OVCa cells in response to miR-551b. Consistent with our in vitro observations, inhibitions of Foxo3 and TRIM31 by miR-551b were observed in mouse xenografts (Fig. 5), suggesting the occurrence of the signaling in vivo.

In summary, our results demonstrate that miR-551b is significantly higher in OVCa SP cells than the non-SP cells. Its upregulation correlates with increased proliferation and invasion of the SP cells in vitro and growth of the mouse xenografts in vivo. Moreover, our data also suggest that miR-551b contributes to the development of chemoresistance of the SP cells in vitro and in vivo, and its inhibition sensitizes cancer cells to chemotherapy, emphasizing its value in future therapeutic development. In an attempt to explore the mechanism of miR-551b functioning, we have shown that miR-551b functions through Foxo3 and TRIM31. However, further studies are required to define the mechanism that triggers its elevation in cancer and the network through which this microRNA relays its signals in its pro-cancerous functions.

## Electronic supplementary material

Below is the link to the electronic supplementary material.

**Supplemental Fig.** **1** miR-551b promotes OVCa cell proliferation and invasion by targeting Foxo3 and TRIM31. The OVCa SP cells were transfected with miR-551b in the presence and absence of Foxo3 or TRIM31 overexpression. The expression of Foxo3 and TRIM31 was confirmed by Western blot (A). The proliferation of the cells was examined by a CCK-8 assay (B, C). Cell cycle distribution of transfected OC cell was profiled by flow cytometry (D). The invasion of cells was determined by using transwell system coated with Matrigel, and the cells migrated were stained with DAPI and imaged (E, Left, 40 × magnification). Cell invasion data from three experiments are summarized in E (right), and presented as mean ± standard deviation. a p < 0.05; b p < 0.01 vs the scramble control; c p < 0.05 vs the miR-551b mimic-treated only. (TIFF 19012 kb)
