# Supplementary figures and images for: Downregulation of Foxo3 and TRIM31 by miR-551b in side population promotes cell proliferation, invasion, and drug resistance of ovarian cancer
Source: Med Oncol. 2016 Oct 14;33(11):126. doi: 10.1007/s12032-016-0842-9 (PMC5065596; doi:10.1007/s12032-016-0842-9)

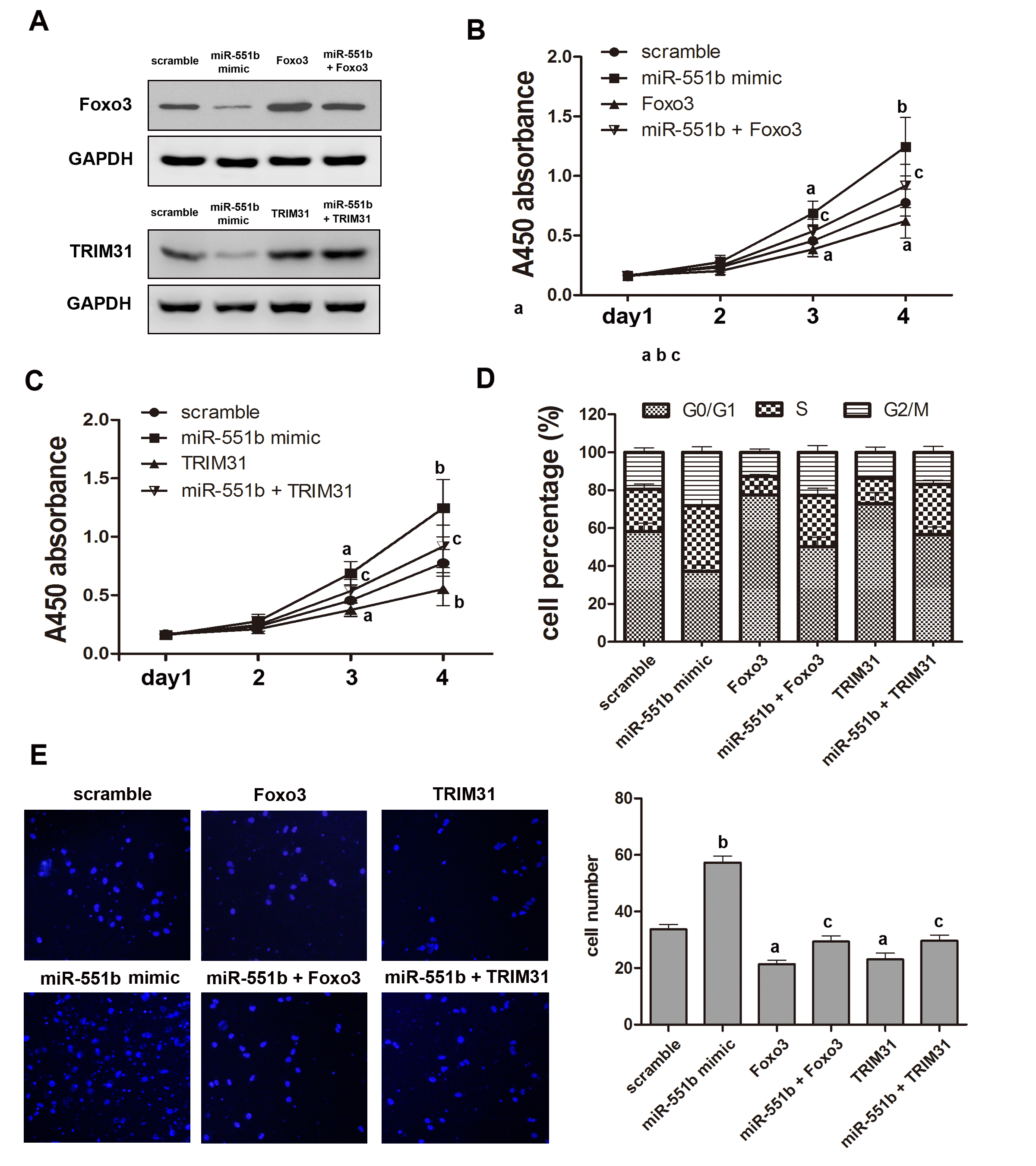

Supplement: Supplementary file 1 — Supplemental Fig. 1 miR-551b promotes OVCa cell proliferation and invasion by targeting Foxo3 and TRIM31. The OVCa SP cells were transfected with miR-551b in the presence and absence of Foxo3 or TRIM31 overexpression. The expression of Foxo3 and TRIM31 was confirmed by Western blot (A). The proliferation of the cells was examined by a CCK-8 assay (B, C). Cell cycle distribution of transfected OC cell was profiled by flow cytometry (D). The invasion of cells was determined by using transwell system coated with Matrigel, and the cells migrated were stained with DAPI and imaged (E, Left, 40 × magnification). Cell invasion data from three experiments are summarized in E (right), and presented as mean ± standard deviation. a p < 0.05; b p < 0.01 vs the scramble control; c p < 0.05 vs the miR-551b mimic-treated only. (TIFF 19012 kb) [file 12032_2016_842_MOESM1_ESM.tif]
